# Parenting and child mental health

**DOI:** 10.1080/17571472.2017.1361630

**Published:** 2017-08-10

**Authors:** Rachael Ryan, Christine O’Farrelly, Paul Ramchandani

**Affiliations:** a The Centre for Psychiatry, Department of Medicine, Imperial College London, London, UK; b Central and North West London (CNWL) Foundation NHS Trust, London, UK

**Keywords:** Mental health, parenting, early intervention, child, behaviour

## Abstract

This paper reviews parenting programmes and their effectiveness with families of young children and highlights additional resources for primary care practitioners. Typically, 30% of GP consultations concern child behaviour problems and established behaviour problems can have lasting effects on children’s life chances. These problems can be identified in infancy and toddlerhood.Parenting is a key risk factor in their development and maintenance, yet is also amenable to change. In this paper we consider six parenting programmes that are widely evaluated and/or available in the U.K. and their evidence base . These include two NICE recommended parenting programmes (*Incredible Years* and *Triple P*), which offer tiered and flexible parenting programmes; predominantly for parents of school-age children. We also review *Parent–Infant Psychotherapy*, which is typically for parents of younger children. Fourth is *Family Nurse Partnership*, an intensive programme to support young, first-time mothers. Finally we consider, video feedback programmes which use video to focus in detail on parents’ interactions with their children, including *Video Feedback to Promote Positive Parenting* and *Video Interactive Guidance.* These interventions demonstrate the range of approaches which are being used to intervene early in children’s lives to try to prevent the development of enduring behavioural problems.

**Why this matters to me:**

It is becoming increasingly clear that the origins of many mental health problems lie in childhood. Family factors, including the quality of care that parents provide for their children, can make a huge difference to children’s early life pathways, for better or for worse. Understanding how best to intervene to support parents is a key challenge. In this article, we critically review the most widely used parenting programmes for parents of young children. It is imperative that we judge these early interventions to high standards so that we are offering children the best start in life.

**Key message:**

Parenting programmes offer a means to intercept behaviour problems in early childhood before they become established.

## Mental health problems in young children: A real problem?

Childhood mental health problems can have lasting effects on children’s life chances. Behaviour problems are the most common mental health problem in early childhood, affecting 5–10% of young children [1,2]. Established problems confer risk for a wide range of negative outcomes including school failure, delinquent behaviour, relationship difficulties, mental illness and physical ill health [3–6]. As such the lifelong cost of behaviour problems to children, families, and society is both substantial and far-reaching [7].

An increasing number of studies indicate that the first signals of behaviour problems can appear as early as infancy and toddlerhood [8–11]. While these difficulties may be transient for some children, for others they persist and represent problems of potential clinical significance [9,11–15]. Consequently, interest has grown in identifying the early precursors of behaviour problems and risk factors associated with their stability. Research shows that these tend to include factors that impinge on parent–child relationships such as family disruption, poor couple functioning, parenting distress, maternal psychopathology and lack of social support [9,10,12,16,17].

Parenting is considered a key risk factor in the development of early psychopathology [18]. Low levels of sensitive parenting and greater use of harsh discipline have been causally linked to the development of behavioural problems [19]. Crucially, however, parenting is amenable to change. Moreover, attempts to enhance parenting early on, when the child’s brain and biological systems that underlie mental health are rapidly taking shape [20], are likely to be especially effective from both a clinical and economic perspective [21]. This understanding, that the first years matter for a lifetime, is now reflected across a number of policy reviews and frameworks [22–30], and forms the basis of the important *1001 Critical Days* manifesto [31].

## The importance of primary care in early mental health

Primary care is recognised as a key context within which to work with parents to optimise early mental health [32]. According to NICE [33] about 30% of a typical GP’s child consultations are for behavioural problems (see Centre for Mental Health for information on presentation https://www.centreformentalhealth.org.uk/childhood-behaviour-briefings [34]). When shared with a GP, parental concerns tend to improve recognition of mental health problems [35], and often identify those children with the most severe problems [36]. Even still, some children will go unidentified. GP access to education about child development and mental health (for example, www.minded.org.uk [37]) and appropriate screening tools (see Szaniecki & Barnes for overview [38]) are likely to be helpful [39]. Information regarding referral options is also key to the integration of early mental health into primary care, especially as less than half of children (5–11-year olds) identified with problems receive referrals for further support [40].

## Parenting programmes as prevention/early intervention

Programmes targeting parenting are the leading early intervention strategy for child behaviour problems. Those designed for preschool and school-aged children with early or established behaviour problems are typically rooted in social learning theory [41,42], which focuses on the cycle whereby caregivers inadvertently reinforce their child’s difficult behaviours, which provokes a negative reaction in the caregiver, and so on, until either the child or caregiver gives in. Interventions based on social learning approaches seek to improve parents’ ability to manage their child’s behaviour by praising/rewarding positive behaviour, setting appropriate limits and applying consistent consequences for undesirable or unwanted behaviour.

*Social learning theory* suggests that children’s behaviour is shaped by the behaviour they observe in their caregivers. It further suggests that caregivers’ responses to children’s behaviour influence the likelihood that children will behave this way more or less frequently in the future. Coercion theory extends these ideas to the use of harsh or physical discipline strategies to propose that they give rise to exchanges that reinforce and escalate aggressive and disruptive behaviour.

Programmes targeting younger at-risk infants and toddlers tend to be more strongly informed by attachment theory which focuses on how the parent–child relationship influences children’s development [43–45]. Attachment interventions are concerned more with the parent’s ability to react in a sensitive way to their child, in terms of their ability to notice, interpret and respond appropriately to their child’s signals. Some approaches of this kind will also include a focus on a parent’s own previous relationships with their parents or carers. Common to both social learning theory and attachment-based programmes, is the premise of improving children’s outcomes by supporting parents in how they relate to and interact with their child on a moment-by-moment basis [46].

*Attachment theory* suggests that children are predisposed to form a strong emotional and physical attachment to at least one primary caregiver. This bond helps children to control negative emotions in times of challenges and stress, develops better social skills, be more confident in exploring the environments around them and acts as the foundation for children’s relationships with others.

There is an extensive evidence base [33,47] for parenting programmes based on social learning principles in improving parenting practices and child behaviour for 3–10-year-old children, at least in the short term[46]. For younger children, a meta-analysis of attachment-based programmes found that those programmes that are brief and have a clear behavioural focus are especially effective in improving parental sensitivity and children’s attachment [48].

Some of the most widely available programmes in the U.K. are described briefly below and summarised in Table 1. These programmes are provided in a range of settings, including through the health visitor led *Healthy Child Programme*, [25] and other integrated children’s services, including in local council services and Children’s Centres and hubs. The importance of early mental health for child outcomes is also reflected in the recent formulation of a specialist health visitor role for perinatal and infant mental health. These specialists provide additional parent–infant relationship supports in complex cases, as well as acting as a key point of contact to GPs, social care and mental health services for families requiring coordinated care [49]. However, only a small minority of health visiting services include these specialist roles.

**Table 1.** Overview of widely available/evaluated parenting programmes.

Programme name	Key characteristics	Population	Key evidence and comments
**Incredible years**	Level of support: universal – targeted tier approach dependent on need	IY Baby Programme: 0–12 months	**IY <3 years**
	Number of sessions: 12–20 weekly sessions	Toddler Basic Programme: 1–3 years	Type of evidence: small-scale RCT
	Location of sessions: in community venues or at home	Preschool Basic Programme: 3–6 years	Parent outcomes: positive treatment effects on parental mental well-being, observed praise and parental depression
	Format of sessions: group or one to one		Child outcomes: positive effects on child development in short term
	Manualised: yes		**IY general:**
	Sessions focus on: strengthening parent–child interactions, nurturing relationships, reducing harsh discipline, and nurturing parents’ ability to promote children’s social, emotional, and language development. In the preschool programme parents also learn how to encourage school readiness skills and work with teachers children’s academic/social skills and emotional literacy		Type of evidence: meta-analysis (age range 3–9 years)
			Parent outcomes: home-based model particularly effective for high risk parents or parents experiencing other interpersonal or health factors
			Child outcomes: effective increasing pro-social behaviour immediately post intervention
**Triple P**	**Baby TP:** 4 group sessions (2 h), 4 weekly telephone consultations (30 mins)	Baby Triple P: 0–12 months	**BTP <1 years**
	**General TP:**	Triple P: 0–16 years	Type of evidence: small-scale RCT
	Level of support: universal – targeted tier approach dependent on need		Parent outcomes: no evidence that BTP improved the quality of the mother-very preterm infant relationship, maternal attachment or responsiveness
	Number of sessions: variable dependent on level		Child outcomes: not measured yet (study in progress)
	Session length: dependent on type of session		**Triple P general:**
	Location of sessions: group delivered in community venues, or one to one at home		Type of evidence: meta-analysis
	Format of sessions: online, individual and group		Parent outcomes: positive treatment effects on parenting practices
	Manualised: yes		Child outcomes: positive treatment effects on children’s behaviour
	Sessions focus on: promoting positive relationships, encouraging desirable behaviours, teaching new skills/behaviours and managing misbehaviour		Notes: results confounded by lack of replication and risk of potential reporting bias
**The Family Nurse Partnership (Building Blocks Trial)**	Level of support: targeted	Nulliparous pregnant women aged 19 or under, recruited by 24 weeks gestation to 2 years old	Type of evidence: adapted from US evidence base. Large-scale RCT
	Number of sessions: up to 64 (14 in pregnancy, 28 from birth to first birthday, 22 between 1 and 2 years)		Parent outcomes: no significant impact on primary outcomes such as pre-natal tobacco use or subsequent pregnancy within 24 months. Positive treatment effects on secondary outcomes including ,self-reported self-efficacy, social support, and partner relationship
	Session length: ~ 1 h 15 min		Child outcomes: no significant impact on primary outcomes including increased birth weight, reduction in number of A&E attendances/hospital admissions. Positive treatment effects on some secondary outcomes including cognitive (maternal-report) and language (maternal report and standardised assessment) development
	Location of sessions: home visits		
	Format of sessions: one to one delivered by trained family nurses		
	Manualised: yes		
	Sessions focus on: positive parent–infant relationships and understanding the baby’s needs, supporting parents in making positive lifestyle choices, increasing parental self-efficacy and ability to build positive relationships with their support networks and access to health and social services		
**Parent infant psychotherapy**	Level of support: targeted	Parents of children who can start antenatal – 2 years	Type of evidence: systematic review
	Number of sessions: ≤49		Parent outcomes: no reliable evidence of benefits for parental sensitivity or depression
	Session length: vary in duration		Child outcomes: suggested improvement in infant attachment security
	Location of sessions: clinic or home based		Notes: improvements in attachment security were derived from low quality studies. There is no evidence of the benefit of PIP over other treatment interventions, such as psycho-educational interventions and cognitive behaviour therapy.
	Format of sessions: one to one		
	Manualised: yes		
	Sessions focus on: improving infant attachment through increasing maternal sensitivity and supporting the parent to reflect on the representations she has of herself as a parent (these are influenced by how they were parented themselves)		
**Video interactive guidance**	Level of support: targeted	Children 0–8 years	Type of evidence: 1 Dutch RCT
	Number of sessions: ~3		Parent outcomes: positive treatment effects for sensitive behaviour and less withdrawn behaviour in mothers but not intrusive behaviour. Positive effects on parental bonding, especially for fathers, but no effects on parental stress and well-being
	Session length: ~2 h		Child outcomes: not reported
	Location of sessions: home based		
	Format of sessions : one to one		
	Manualised: no		
	Sessions focus on: filmed and edited parent–child interactions are reviewed to promote positive interactions; particularly moments when the adult has responded in an appropriate way to the child’s action using both verbal and non-verbal communication		
**Video feedback intervention to promote positive parenting**	Level of support: targeted	Children:4–47 months	Type of evidence: meta-analysis
	Number of sessions: 4–6		Parent outcomes: strong evidence base of increased parental sensitivity and positive parenting across various populations
	Session length: 1 h		Child outcomes: positive treatment effects on attachment and problem behaviour
	Location of sessions: home based		Notes: strong replication of evidence across countries and cohorts
	Format of sessions: one to one		
	Manualised: yes		
	Sessions focus on: unedited recordings of parent–child interactions are reviewed. Core themes are used to provide structured positive feedback to promote sensitive responding. Themes include exploration and attachment, non-coercive discipline, use of positive/negative reinforcement, distracting and postponing, responding sensitively to signals, sensitive time out and empathy		

## Incredible years

The Webster-Stratton *Incredible Years* intervention is a prominent suite of programmes for parents of children aged 0–12 years, and is NICE recommended for children with conduct disorder. Grounded in social learning theory, the interventions vary over five levels of intensity depending on need. The series uses video vignettes and role play to discuss parents’ use of play skills, praise and rewards, limit setting and strategies for handling misbehaviour. A recent meta-analysis of 50 studies involving children (mean age 3–9 years) showed a small effect size across informants, with larger effects shown for children with the most severe problems [50]. Studies examining the programme’s effectiveness with young toddlers (1–2 years) demonstrated significant improvements in parental mental well-being and praise [51], and parent behaviour was improved in studies with older toddlers (2–3 years), however, effects were not observed for child behaviour problems [52–54]. Thus, whilst there is some evidence for the effectiveness of these programmes in school-age children, there is at present still little evidence for an effect on behaviour in younger children [55].

## Triple P positive parenting program

The *Triple P Positive Parenting Programs*, developed in Australia by Sanders [56] are also informed by social learning theory. They aim to prevent emotional and behavioural problems in children aged 0–16 by building parents’ knowledge, skills and confidence. Also recommended by NICE, *Triple P* seeks to engage both mothers and fathers across five levels/intensities, based on the severity of child behaviour and the family’s level of need, through both universal and targeted approaches. At a low intensity, parents attend group seminars, while higher intensity formats typically include more individual supports for at-risk parents or those with specific concerns. Systematic reviews, however, show mixed evidence for the intervention’s effectiveness [57–59]. Additionally, trials evaluating *Baby Triple P*, which targeted mothers with postnatal depression and pre-term infants, have shown no treatment effects on parent–child outcomes [60,61].

## Parent–infant psychotherapy

Originally developed in the US by Selma Fraiberg (1980), *Parent–Infant Psychotherapy (PIP)* aims to support and restore the parent–infant relationship by working with the parent infant dyad. *PIP* varies in its delivery, but most models take their roots from psychoanalysis and aspects of attachment theory [62]. Sessions typically take place with both the parent and infant together and involve the psychotherapist observing the parent–infant interaction, listening to and identifying concerns and worries and supporting the parent to develop different ways to relate to their infant [63]. Emphasis is placed on parents’ internal working models or representations of the infant in the context of their own caregiving history. A recent Cochrane review [63] identified only weak evidence for *PIP*; the few effects found for infant attachment in high-risk populations were based on low quality studies and there was no evidence of *PIP*’s effectiveness when compared to other interventions. More recent studies have also failed to demonstrate effects for infant outcomes [64].

## Family nurse partnership

More intensive initiatives targeting young parents include The *Family Nurse* *Partnership* (FNP) [65]. *FNP* works with young (≤19 years old) first time mothers to provide support from early pregnancy until the child is aged 2 years. Originating from an evidence-based model developed in the US [66], families receive up to 64 home visits from trained family nurses. Visits target prenatal health-related behaviours, sensitive and competent parenting and maternal self-sufficiency through a number of core topics. Topics include personal and environmental health, life course development, the maternal role, family and friends and access to health and social services. Family nurses seek to effect maternal behaviour change by enhancing maternal self-efficacy. They also adopt a strengths-based approach to education and modelling activities to promote sensitive and competent caregiving and reduce the risk of maltreatment [67]. A rigorous multi-site evaluation of *FNP* in the U.K. found high levels of maternal engagement, yet no benefits on pre-defined child and maternal outcomes including smoking cessation, birth weight, subsequent pregnancies and emergency hospital admissions [68]. A longer term follow up is underway, which may inform whether any additional benefits accrue over time. However, some programmes have since been decommissioned.

## Video feedback interventions

Interest is also growing in interventions that utilise video feedback methods as a means of promoting young children’s behaviour through increased parental sensitivity. These interventions typically involve filming parents and infants together during different situations (e.g. playing together and mealtimes) which are then reviewed with a therapist to highlight moments of positive interaction. There are several different forms that this therapy can take – the two most often studied or used in the U.K. both originate in the Netherlands, and are known as *Video Feedback to Promote Positive Parenting (VIPP)* and *Video Interaction Guidance (VIG).*

*VIG* involves the co-construction of goals by the parent and therapist with the aim of promoting a harmonious and responsive parent–child relationship through enhanced two-way communication. Under the *VIG* model, the therapist uses feedback on a few selected clips of ‘better than usual’ parent–child interaction that capture successful moments of interaction, to encourage the parent to reflect on what is going well and to promote further instances of sensitivity and attunement. However, there has been limited evaluation of *VIG* [69,70]. While a recent study of preterm infants found a benefit for parental sensitivity, no effects were found for child outcomes [71]. To date in the U.K., there has only been a small pilot RCT and so there is limited data on which to judge its effectiveness [72].

The majority of video feedback research is based on trials of a different intervention – *VIPP* [73], which was developed by researchers at Leiden University. *VIPP* is a brief, manualised, home-based intervention involving six visits that aim to promote parent–child relationships by enhancing sensitive parenting and also focusing on strategies for managing difficult behaviour. *VIPP* is based on a combination of attachment theory [45] and coercion theory, a version of social learning theory [42]. At each visit, prepared feedback is given by the therapist as the parent and therapist review video clips together, using positive comments based on the intervention’s themes. These comments are used to support the parent in perceiving and interpreting their child’s behaviour, emotions and expressions and to respond to these signals in a sensitive way [74]. A strong body of evidence from a wide range of countries and families supports the effectiveness of *VIPP* in promoting maternal sensitivity [74–76], although the evidence for effects on children’s behaviour remains limited [75–79].

Current U.K. research is underway at Imperial College London to evaluate the effectiveness and cost-effectiveness of *VIPP* in preventing enduring behavioural problems in children aged 12–36 months old. This National Institute for Health Research funded research takes the form of a randomised controlled trial of 300 families, and is recruiting from primary care and community settings across several boroughs in London (Hillingdon, Camden, Islington and Barking and Dagenham). This will give a rigorous assessment of the effectiveness and cost-effectiveness of this intervention in usual NHS practice.

In summary, there is mixed evidence across parenting interventions for school-age and older preschool children, with some programmes, such as the *Incredible Years*, demonstrating a strong evidence base [46]. There is more limited evidence for programmes that target families and children in the first two years of life, thus forthcoming research, such as the *FNP* follow up study and *VIPP* trial, are likely to be instructive in this regard. Further work is also needed to better identify what type and intensity of programme is likely to be most effective and cost-effective for families with different needs and preferences. Insight is also needed on the pathways that underlie programme effects and whether and how long we can expect these outcomes to be sustained.

Parenting programmes are also likely to be most effective when early mental health is promoted in the wider systems and contexts that surround children and their families. This often necessitates innovative and collaborative ways of working across NHS, local authority and community services (e.g. early childhood care and education settings, schools, multidisciplinary child development teams, child and adolescent mental health services and social care), as can be seen in recent examples in school settings [80,81]. Primary care practitioners are a lynch pin in this work, as they see daily examples of the role that early experiences and relationships play in shaping the foundations of later health [81]. Through early identification and appropriate referral, primary care settings can help families to develop the relationships that offer children the best start in life and reduce the burden of mental health problems for families and society [82].

*Additional resources:*

• *More information about your local Children’s Centre can be found* at https://childrenscentresfinder.direct.gov.uk/childrenscentresfinder
• *Additional training for GPs in child development and mental health can be found at* www.minded.org.uk
• *Data on prevalence and risk factors, divided by area, can be found* using The Children and Young People’s Mental Health and Wellbeing Profiling Tool at https://fingertips.phe.org.uk/profile-group/child-health
• *Referral information for parenting interventions (school age children) can be found at* https://webarchive.nationalarchives.gov.uk/20140311170415/https:/www.education.gov.uk/commissioning-toolkit/Programme/CommissionersSearch
• *A review of the evidence and cost of relevant early intervention programmes by the Early Intervention Foundation can be found here* https://www.eif.org.uk/publication/foundations-for-life-what-works-to-support-parent-child-interaction-in-the-early-years/

## Disclosure statement

Paul Ramchandani receives funding from the National Institute of Health Research (NIHR) to develop and evaluate early interventions to promote infant mental health in early life, including a randomised controlled trial of VIPP for children at risk of behavioural problems.
